# 2,5-Dichloro-3,6-diisopropyl­cyclo­hexa-2,5-diene-1,4-dione

**DOI:** 10.1107/S1600536812032886

**Published:** 2012-08-11

**Authors:** Ping Li, Hai Wang, Jian Dong, Hong-Yu Chen

**Affiliations:** a Faculty of Chemistry and Chemical Engineering, TaiShan Medical University, Tai’an 271016, People’s Republic of China

## Abstract

The mol­ecule of the title compound, C_12_H_14_Cl_2_O_2_, lies about an inversion center. The six-membered ring is almost planar, with the largest deviation from the least-squares plane being 0.014 (4) Å. The mol­ecular conformation is stabilized by a weak intra­molecular C—H⋯O hydrogen bond. In the crystal, mol­ecules are packed into stacks along the *c*-axis direction, with an inter­centroid separation of 4.811 (2) Å. Neighboring mol­ecules within the stack are related by the *c*-glide plane.

## Related literature

Metal complexes of catechols, semiquinones and quinones are of general inter­est in the investigation of ligand centered redox reactions and as models for biochemical processes involving metal ions, see: Mostafa (1999 ▶). For standard bond lengths, see: Allen *et al.* (1987 ▶).
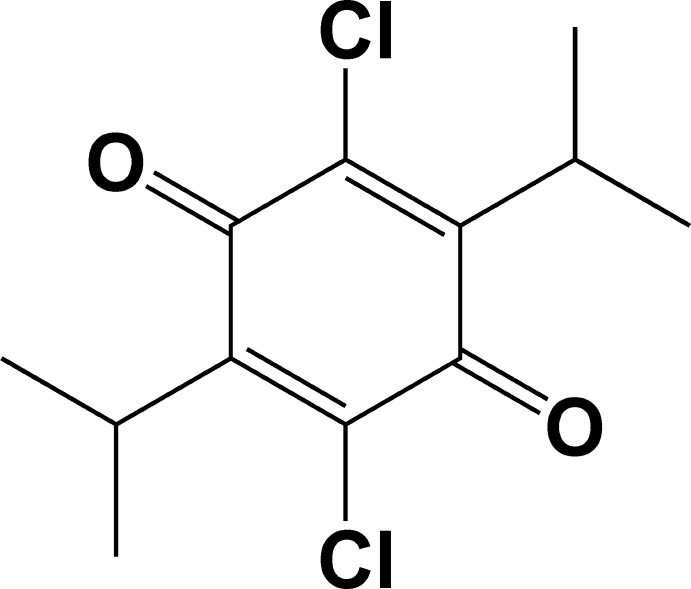


## Experimental

### 

#### Crystal data

C_12_H_14_Cl_2_O_4_
*M* *_r_* = 293.13
Monoclinic, 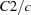

*a* = 10.286 (2) Å
*b* = 15.034 (3) Å
*c* = 9.621 (2) Å
β = 109.022 (4)°
*V* = 1406.5 (6) Å^3^
*Z* = 4
Mo *K*α radiation
μ = 0.46 mm^−1^
*T* = 293 K
0.15 × 0.13 × 0.12 mm

#### Data collection

Brucker APEXII CCD diffractometer
Absorption correction: multi-scan (*SADABS*; Bruker, 2007 ▶) *T*_min_ = 0.925, *T*_max_ = 0.946
3544 measured reflections
1248 independent reflections
676 reflections with *I* > 2σ(*I*)
*R*_int_ = 0.079

#### Refinement

*R*[*F* ^2^ > 2σ(*F* ^2^)] = 0.059
*wR*(*F* ^2^) = 0.198
*S* = 1.08
1248 reflections
84 parameters
12 restraints
H-atom parameters constrained
Δρ_max_ = 0.41 e Å^−3^
Δρ_min_ = −0.24 e Å^−3^

### 

Data collection: *APEX2* (Bruker, 2007 ▶); cell refinement: *SAINT* (Bruker, 2007 ▶); data reduction: *SAINT*; program(s) used to solve structure: *SIR97* (Altomare *et al.*, 1999 ▶); program(s) used to refine structure: *SHELXL97* (Sheldrick, 2008 ▶); molecular graphics: *SHELXTL* (Sheldrick, 2008 ▶); software used to prepare material for publication: *WinGX* (Farrugia, 1999 ▶).

## Supplementary Material

Crystal structure: contains datablock(s) global, I. DOI: 10.1107/S1600536812032886/yk2064sup1.cif

Structure factors: contains datablock(s) I. DOI: 10.1107/S1600536812032886/yk2064Isup2.hkl

Supplementary material file. DOI: 10.1107/S1600536812032886/yk2064Isup3.cml

Additional supplementary materials:  crystallographic information; 3D view; checkCIF report

## Figures and Tables

**Table 1:** Hydrogen-bond geometry (Å, °)

*D*—H⋯*A*	*D*—H	H⋯*A*	*D*⋯*A*	*D*—H⋯*A*
C4—H4⋯O1^i^	0.98	2.34	2.926 (7)	117

Symmetry code: (i) 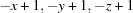.

**Fig. 1.**
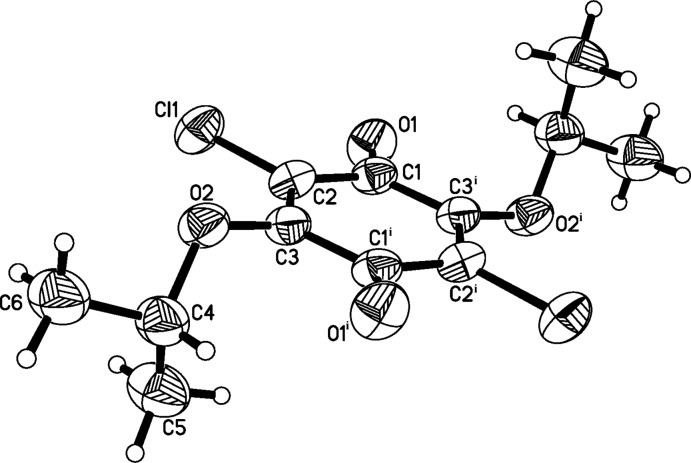
Molecular structure with atom labelling scheme and thermal ellipsoids drawn at the 30% probability level (symmetry code (i): 1 - x,1 - y,1 - z).

## supplementary crystallographic information

### Comment

Metal complexes of catechols, semiquinones and quinones are of general interest in the investigation of ligand centered redox reactions and as models for biochemical processes involving metal ions (Mostafa, 1999). The title compound is the synthetic precusor for chloranilic acid, which is a simple, readily available ligand combining chelating and bridging capabilities.

In the title molecule, the six-membered ring and attached oxygen and clorine atoms bound to every vertex of this carbon hexagon, share a same plane with the largest deviation being 0.053 (4) Å for C3. The two isopropyl groups extend from the plane, one above and one below the plane, as shown in Fig. 1. The C1═O1 bond has a lengths of 1.221 (4) Å, typical of C*sp*^2^═ O double bonds (Allen *et al.*, 1987). The C3—O2 bond, however, is a C*sp*^2^—O single bond with the lengths of 1.346 (5) Å, which is slightly shorter than the value expected for enol ester systems [1.354 (16) Å (Allen *et al.*,1987). The carbon-carbon bonds in the six-membered ring can also be divided into two groups: the C2═C3 bond is a typical double bond with the length of 1.343 (5) Å, whereas the C1—C2 and C1—C3^i^ bonds with the lengths of 1.463 (5) Å and 1.480 (6) Å, reaspectively, are obviously the C*sp*^2^—C*sp*^2^ single bonds.

There are weak intramolecular interactions C2—H2A···O2 (1 - *x*,1 - *y*,1 - *z*) [H···O = 2.34 Å, C···O = 2.926 (7) Å, and C—H···O = 117°], which stabilize the molecule conformation.

In the crystal, the molecules of the title compound are packed into stacks along the *c* direction with intercentroid separation of 4.811 (2) Å. Neighboring molecules within the stack are related by the *c* glide plane.

### Experimental

Potassium hydroxide (5.0 g) was added to a solution containing chloranil (5.0 g) in 2-propanol (100 ml). The resulting mixture was stirred under reflux for 1 h, and then the red reaction solution was cooled to 283 K. The precipitated yellow solid was collected and recrystallized in ethanol.

### Refinement

All H atoms were positioned geometrically and allowed to ride on their parent atoms with C—H = 0.98 Å and *U*_iso_(H)= 1.2*U*_eq_(C) for tertiary hydrogen and with C—H = 0.96 Å and *U*_iso_(H)= 1.5*U*_eq_(C) for methyl group.

### Crystal data

**Table:**

C_12_H_14_Cl_2_O_4_	*F*(000) = 608
*M**_r_* = 293.13	*D*_x_ = 1.384 Mg m^−^^3^
Monoclinic, *C*2/*c*	Mo *K*α radiation, λ = 0.71069 Å
Hall symbol: -C 2yc	Cell parameters from 837 reflections
*a* = 10.286 (2) Å	θ = 2.5–21.5°
*b* = 15.034 (3) Å	µ = 0.46 mm^−^^1^
*c* = 9.621 (2) Å	*T* = 293 K
β = 109.022 (4)°	Block, yellow
*V* = 1406.5 (6) Å^3^	0.15 × 0.13 × 0.12 mm
*Z* = 4	

### Data collection

**Table:**

Brucker APEXII CCD diffractometer	1248 independent reflections
Radiation source: fine-focus sealed tube	676 reflections with *I* > 2σ(*I*)
Graphite monochromator	*R*_int_ = 0.079
φ and ω scans	θ_max_ = 25.0°, θ_min_ = 2.5°
Absorption correction: multi-scan (*SADABS*; Bruker, 2007)	*h* = −12→9
*T*_min_ = 0.925, *T*_max_ = 0.946	*k* = −17→17
3544 measured reflections	*l* = −10→11

### Refinement

**Table:**

Refinement on *F*^2^	Primary atom site location: structure-invariant direct methods
Least-squares matrix: full	Secondary atom site location: difference Fourier map
*R*[*F*^2^ > 2σ(*F*^2^)] = 0.059	Hydrogen site location: inferred from neighbouring sites
*wR*(*F*^2^) = 0.198	H-atom parameters constrained
*S* = 1.08	*w* = 1/[σ^2^(*F*_o_^2^) + (0.1*P*)^2^ + 0.*P*] where *P* = (*F*_o_^2^ + 2*F*_c_^2^)/3
1248 reflections	(Δ/σ)_max_ = 0.001
84 parameters	Δρ_max_ = 0.41 e Å^−^^3^
12 restraints	Δρ_min_ = −0.24 e Å^−^^3^

### Special details

**Table:**

Geometry. All s.u.'s (except the s.u. in the dihedral angle between two l.s. planes) are estimated using the full covariance matrix. The cell s.u.'s are taken into account individually in the estimation of s.u.'s in distances, angles and torsion angles; correlations between s.u.'s in cell parameters are only used when they are defined by crystal symmetry. An approximate (isotropic) treatment of cell e.s.d.'s is used for estimating s.u.'s involving l.s. planes.
Refinement. Refinement of *F*^2^ against ALL reflections. The weighted *R*-factor *wR* and goodness of fit *S* are based on *F*^2^, conventional *R*-factors *R* are based on *F*, with *F* set to zero for negative *F*^2^. The threshold expression of *F*^2^ > σ(*F*^2^) is used only for calculating *R*-factors(gt) *etc*. and is not relevant to the choice of reflections for refinement. *R*-factors based on *F*^2^ are statistically about twice as large as those based on *F*, and *R*- factors based on ALL data will be even larger.

### Fractional atomic coordinates and isotropic or equivalent isotropic displacement parameters (Å^2^)

**Table:**

	*x*	*y*	*z*	*U*_iso_*/*U*_eq_	
C1	0.4914 (5)	0.5935 (3)	0.5241 (4)	0.0672 (11)	
C2	0.5842 (4)	0.5580 (3)	0.4504 (4)	0.0686 (12)	
C3	0.5968 (4)	0.4704 (3)	0.4308 (4)	0.0632 (11)	
C4	0.7787 (5)	0.3702 (3)	0.4212 (5)	0.0903 (13)	
H4	0.7285	0.3149	0.4223	0.108*	
C5	0.8654 (6)	0.3916 (4)	0.5744 (6)	0.1146 (16)	
H5A	0.9196	0.4436	0.5740	0.172*	
H5B	0.9252	0.3424	0.6150	0.172*	
H5C	0.8075	0.4027	0.6331	0.172*	
C6	0.8576 (7)	0.3595 (4)	0.3175 (6)	0.1114 (16)	
H6A	0.7949	0.3508	0.2198	0.167*	
H6B	0.9173	0.3089	0.3459	0.167*	
H6C	0.9115	0.4120	0.3198	0.167*	
Cl1	0.68016 (14)	0.63397 (8)	0.39265 (14)	0.0914 (7)	
O1	0.4891 (4)	0.6728 (2)	0.5509 (4)	0.0948 (11)	
O2	0.6789 (3)	0.4409 (2)	0.3567 (3)	0.0789 (10)	

### Atomic displacement parameters (Å^2^)

**Table:**

	*U*^11^	*U*^22^	*U*^33^	*U*^12^	*U*^13^	*U*^23^
C1	0.085 (3)	0.052 (3)	0.065 (3)	0.012 (2)	0.025 (2)	0.007 (2)
C2	0.086 (3)	0.061 (3)	0.058 (2)	−0.001 (2)	0.022 (2)	0.007 (2)
C3	0.075 (3)	0.059 (3)	0.057 (2)	0.010 (2)	0.023 (2)	0.0061 (19)
C4	0.095 (3)	0.100 (3)	0.079 (3)	0.027 (2)	0.033 (2)	0.006 (2)
C5	0.114 (3)	0.124 (3)	0.094 (3)	0.026 (3)	0.018 (3)	−0.006 (3)
C6	0.124 (3)	0.121 (3)	0.092 (3)	0.044 (3)	0.040 (3)	0.004 (3)
Cl1	0.1161 (12)	0.0720 (9)	0.0961 (10)	−0.0080 (6)	0.0484 (8)	0.0120 (6)
O1	0.125 (3)	0.055 (2)	0.119 (3)	0.0052 (18)	0.059 (2)	−0.0032 (18)
O2	0.102 (2)	0.0689 (19)	0.075 (2)	0.0238 (16)	0.0414 (18)	0.0111 (14)

### Geometric parameters (Å, º)

**Table:**

C1—O1	1.221 (4)	C4—C5	1.489 (7)
C1—C2	1.463 (5)	C4—H4	0.9800
C1—C3^i^	1.480 (6)	C5—H5A	0.9600
C2—C3	1.343 (5)	C5—H5B	0.9600
C2—Cl1	1.715 (4)	C5—H5C	0.9600
C3—O2	1.346 (5)	C6—H6A	0.9600
C4—O2	1.468 (5)	C6—H6B	0.9600
C4—C6	1.486 (7)	C6—H6C	0.9600
			
O1—C1—C2	121.2 (4)	C5—C4—H4	108.7
O1—C1—C3^i^	121.0 (4)	C4—C5—H5A	109.5
C2—C1—C3^i^	117.7 (4)	C4—C5—H5B	109.5
C3—C2—C1	122.2 (4)	H5A—C5—H5B	109.5
C3—C2—Cl1	121.1 (3)	C4—C5—H5C	109.5
C1—C2—Cl1	116.7 (3)	H5A—C5—H5C	109.5
C2—C3—O2	120.2 (4)	H5B—C5—H5C	109.5
C2—C3—C1^i^	120.0 (4)	C4—C6—H6A	109.5
O2—C3—C1^i^	119.5 (4)	C4—C6—H6B	109.5
O2—C4—C6	104.7 (4)	H6A—C6—H6B	109.5
O2—C4—C5	111.9 (4)	C4—C6—H6C	109.5
C6—C4—C5	114.0 (5)	H6A—C6—H6C	109.5
O2—C4—H4	108.7	H6B—C6—H6C	109.5
C6—C4—H4	108.7	C3—O2—C4	119.2 (3)
			
O1—C1—C2—C3	174.3 (4)	C1—C2—C3—C1^i^	4.0 (6)
C3^i^—C1—C2—C3	−3.9 (6)	Cl1—C2—C3—C1^i^	−177.4 (3)
O1—C1—C2—Cl1	−4.3 (5)	C2—C3—O2—C4	130.7 (4)
C3^i^—C1—C2—Cl1	177.4 (3)	C1^i^—C3—O2—C4	−56.2 (5)
C1—C2—C3—O2	177.1 (3)	C6—C4—O2—C3	−175.7 (4)
Cl1—C2—C3—O2	−4.4 (5)	C5—C4—O2—C3	−51.8 (6)

Symmetry code: (i) −*x*+1, −*y*+1, −*z*+1.

### Hydrogen-bond geometry (Å, º)

**Table:**

*D*—H···*A*	*D*—H	H···*A*	*D*···*A*	*D*—H···*A*
C4—H4···O1^i^	0.98	2.34	2.926 (7)	117

Symmetry code: (i) −*x*+1, −*y*+1, −*z*+1.
